# A130 LONGITUDINAL GROWTH OF PREESCHOOLERS WITH EOSINOPHILIC ESOPHAGITIS

**DOI:** 10.1093/jcag/gwab049.129

**Published:** 2022-02-21

**Authors:** E Carrión-Jaramillo, M Vurzinger, V Avinashi

**Affiliations:** 1 Pediatric Gastroenterology, BC Children’s Hospital, Vancouver, BC, Canada; 2 BC Children’s Hospital, Vancouver, BC, Canada

## Abstract

**Background:**

Eosinophilic esophagitis (EoE) is an immune-mediated condition involving eosinophils infiltrating the esophagus. While adults predominantly present with dysphagia, infants and preschoolers can present with vomiting, poor solid food progression, and oral aversion. Growth is a concern at diagnosis as well as in follow-up based on poor intake, prescribed food elimination, and the potential impact of swallowed steroids.

**Aims:**

Assess baseline anthropometric measurements in preescholers with EoE and evaluate their growth parameters in follow up.

**Methods:**

A retrospective chart review of patients with EoE at BC Children’s Hospital from 2007–21. Inclusion criteria were patients diagnosed before 5 years, with *>* 2 anthropometrics measurements. Variables included weight, height, BMI, and Z-scores (CDC >2 years; WHO <2 years). The change in Z-scores were calculated between the first registered clinical visit after diagnosis and the most recent growth parameters available. Statistical difference between Z-scores for height and BMI was determined with a paired t-test. Differences in growth according to treatment used one way t-test.

**Results:**

There were 44 patients included. Mean age of diagnosis was 3 years, 82% were male. The average follow up time was 4.9 years (0.5–12.2 years). At baseline, 1 patient was severely malnourished (Z<-3) and 1 was moderately malnourished based on BMI (Z<-2). At follow-up there were 2 patients with moderate malnutrition. The change in BMI Z-scores over time was relatively neutral -0.05±1.3SD but there were 3 patients with a decline in Z-score more than 2, a different way to represent malnutrition.

Stunting was more common at baseline with 3 patients having severe stunting (Z<-3), and 5 having moderate stunting (Z<-2). At follow-up 1 patient had severe stunting BMI (Z<-3), and 1 had moderate stunting (Z<-2). The change in height Z-scores was net +0.41±1.21SD.

Last reported treatments included 45.5% on steroids, 18.2% on elimination diet, 11.4% on steroid/diet, 4.5% on PPI, 4.5% on PPI/diet and 15.9% no treatment. There were no significant differences in BMI Z-scores or height Z-scores according to treatment.

**Conclusions:**

Among those diagnosed with EoE at less than 5 years of age, BMI was stable from diagnosis through follow up and reflected the general population. Stunting was observed in as many as 1/5 of patients at diagnosis but did show improvement in follow up. Further analysis would benefit from a larger sample, further treatment details and correlation with esophageal inflammation.

**Funding Agencies:**

None

**Table:** 

	Baseline	Las reported	p value
Mean age (years)	3.3± 1.6	8.7± 2.9	
BMI%	50.4± 34.5	50.1± 32.4	
BMI Z score (SD)	+0.06± 1.53	+0.02± 1.20	0.8
%Malnutrition Z<-2 BMI	4.5	4.5	
Height% for age	34.8	41.4	
%Stunting Z<-2	18.2	4.5	0.03*

